# Knowledge and experiences of Chagas disease in Bolivian women living in Spain: a qualitative study

**DOI:** 10.3402/gha.v9.30201

**Published:** 2016-03-11

**Authors:** Teresa Blasco-Hernández, Lucía García-San Miguel, Bárbara Navaza, Miriam Navarro, Agustín Benito

**Affiliations:** 1 National Centre for Tropical Medicine, Health Institute Carlos III, Madrid, Spain; 2 Department of Preventive Medicine, Puerta de Hierro Hospital, Majadahonda, Madrid, Spain; 3 Fundación Mundo Sano, Spain

**Keywords:** neglected tropical diseases, Chagas disease, socio-cultural, immigration, qualitative research

## Abstract

**Background:**

In Europe, Spain has the highest number of people with Chagas disease (CD). Bolivian migrants account for 81% of the reported cases. One of the priorities in controlling the disease is prevention of mother-to-child transmission. Despite under-diagnosis in Spain being estimated at 90%, there are currently few studies that explore the social and cultural dimensions of this disease.

**Objective:**

The aim of this study was to explore the knowledge and experiences of Bolivian women with CD, in order to generate a useful understanding for the design and implementation of public health initiatives.

**Design:**

Qualitative study based on semi-structured interviews, triangular groups, and field notes.

**Participants:**

Fourteen Bolivian women with CD living in Madrid.

**Results:**

The participants were aware that the disease was transmitted through the vector, that it could be asymptomatic, and that it could also be associated with sudden death by heart failure. They opined that the treatment as such could not cure the disease but only *slow it down*. There was a sense of indifference along with a lack of understanding of the risk of contracting the disease. Participants who presented with symptoms, or those with relatives suffering from the disease, were concerned about fatalities, cardiac problems, and possible vertical transmission. There was also a fear of being rejected by others. The disease was described as something that affected a large number of people but only *showed up in a few cases and that too after many years*. There was a widespread assumption that it was *better not to know* because doing so, allows *the disease to take hold*.

**Conclusions:**

Disease risk perception was very low in Bolivian women living in Madrid. This factor, together with the fear of being screened, may be contributing to the current rate of under-diagnosis.

## Introduction

Chagas disease (CD) is a parasitic infection caused by *Trypanosoma cruzi* (*T. cruzi*) and is endemic to regions in Latin America. It is transmitted mostly in rural areas by a vector *Triatoma infestans* (*T. infestans*). Transmission via blood transfusion, transplants, and mother to child is also possible. Most people remain asymptomatic after infection and one-thirds will develop chronic heart and/or bowel disease. Treatment with benznidazole or nifurtimox is extremely toxic and has limited effectiveness when the disease is chronic. However, it is effective in the reduction of congenital transmission.

According to the World Health Organization (WHO), there are more than 10 million people infected by *T. cruzi* and the majority live in Latin American countries (1). In Europe, Spain has the largest number of Latin American migrants, more than 2 million (2), which is why it is the European country with the highest number of CD cases (3, 4). According to the available data, there are more than 50,000 people infected by *T. cruzi* in Spain (5) out of which, 60% are women of child-bearing age (3). The estimated average rate of congenital transmission is 4.35% (5–7). Majority of diagnosed Chagas cases in Spain (81%) are amongst the 190,000 Bolivians living in the country (3). The prevalence of infection in this population is estimated to be over 20% (5).

In Spain, all blood products and organs are screened for CD (8, 9). There are also some regional initiatives to screen the other possible mode of transmission: congenital transmission during pregnancy. Nonetheless, with under-diagnosis estimated to be at 90% (3, 5), there is no national plan for the control of CD. Moreover, there are few qualitative studies offering a social and cultural dimension of this disease in immigrants living in Spain (10–12).

The aim of this study is therefore to explore the knowledge and experiences of a group of Bolivian women with CD living in Madrid in order to generate a useful understanding for the design of public health policies adapted to the social and cultural context of the population at risk.

## Design

This is a qualitative study with a constructionist perspective where Grounded Theory has been used to analyse data (13). Grounded Theory is an analytical process based on the symbolic interactionism tradition. It consists of coding the information collected in successive steps and from an inductive perspective in order to create a general theory (13). In this case, we use Grounded Theory to identify knowledge and experiences of Chagas and relate this to explicative variables. Data were gathered through semi-structured interviews; the key questions to be asked were decided on in advance and a detailed interview structure was created. Triangular groups, an alternative to focus groups, were also used in order to merge the individual and the group discourse. Triangular groups are composed of fewer people and their dynamic is less structured than in focus groups (14). Observation field notes were employed as a complementary technique (15).

### Scope of the study

The study was carried out in the Community of Madrid (CM). Part of the research was framed within one of the health programmes created by the Fundación Mundo Sano, which focused on training a group of Bolivian women living in the CM to become community health workers. The study was developed in different public health facilities, administrative centres, and the National Centre for Tropical Medicine, as well as the private homes of the participants.

### Study subjects

Women were selected from different public healthcare centres and via the Association of Friends of People with CD (ASAPECHA in Spanish). The criteria for inclusion were: being a woman from Latin America and to have attended a Chagas screening service.

### Field work

Fourteen interviews were performed in total, as well as two triangular groups (Table 1). Sixteen field notes were collected during the training programme. A summary of the key topics introduced during the course of the interviews and the triangular groups is provided (Table 2).

**Table 1:** Profile of the participants in the study

	Interview no.	Age	Region of origin	Education	Employment status	Public healthcare coverage	No. of children	Years in Spain	Referred by
Participants in	E1	31	Cochabamba	Secondary education	Housekeeper	Yes	2	10	H
the training	E2	31	Cochabamba	Secondary education	Unemployed	Yes	2	10	H
programme	E3	35	Cochabamba	Secondary education	Housekeeper	Yes	2	10	H
	E4	34	Cochabamba	Secondary education	Housekeeper	Yes	1	15	H
	E5	47	Cochabamba	Secondary education	Housekeeper	Yes	3	9	H
	E6	28	Santa Cruz	Secondary education	Housekeeper	Yes	1	6	HC
Other	E7	47	Cochabamba	Secondary education	Housekeeper	Yes	3	9	H
participants	E8	50	Trinidad/Santa Cruz	Secondary education	Unemployed	Yes	5	9	R
	E9	44	Santa Cruz	Secondary education	Beautician	Yes	1	13	HC
	E10	34	Cochabamba/Santa Cruz	Secondary education	Housekeeper	Yes	3	9	HC
	E11	42	Vallegrande/Santa Cruz	Primary education	Housekeeper	Yes	3	7	HC
	E12	45	Cochabamba	Primary education	Housekeeper	Yes	4	9	H
	E13	33	Santa Cruz	Primary education	Housekeeper	Yes	3	10	A
	E14	35	Santa Cruz	Primary education	Housekeeper	Yes	3	9	A
Triangular		31	Cochabamba	Secondary education	Housekeeper	Yes	2	10	H
Group 1		34	Cochabamba	Secondary education	Housekeeper	Yes	1	15	H
		47	Cochabamba	Secondary education	Housekeeper	Yes	3	9	H
Triangular		28	Santa Cruz	Secondary education	Housekeeper	Yes	1	6	HC
Group 2		31	Cochabamba	Secondary education	Housekeeper	Yes	2	10	H
		31	Cochabamba	Secondary education	Unemployed	Yes	2	10	H

A, association; H, hospital; HC, Healthcare centre; R, other participant's relative.

**Table 2:** Main topics included in the interview and group's guidelines

Knowledge and perceptions
Transmission: vector, other transmission routes, possibility of transmission in Spain
Symptoms and diagnosis
Treatment and its side effects

Personal and social experiences

How the diagnosis was performed and the reasons for undergoing the test
The meaning of Chagas disease
The moment of the diagnosis
Experience of the disease and treatment
Consequences of the disease for their own lives and that of others
Social support and people consulted for health-related advice
Social rejection caused by the disease

Disease representation in Spain and in Bolivia

Memories of their lives in Bolivia
Social representations of the disease in Spain

Field work was developed in two stages. The first of these was the period from March to September 2013, during the training course. Once all the data had been analysed in detail, we initiated the second stage of the study, from November 2013 to March 2014, in order to reach data saturation (16).

### Data analysis

Analysis of the data was performed using the OpenCode 3.6 programme of the University of Umea, Sweden. For analysis of the initial interviews, codes of meaning were identified and later grouped into categories which were then developed and applied to the social context of Bolivia and Spain. Through these means, theories surrounding the meanings expressed by the participants were generated. These theories were tested in subsequent interviews and the material obtained in these encounters was again codified and added to the previous analyses.

### Scientific rigour

All research was conducted in Spanish. An external expert transcribed all the interviews. Transcriptions were subjected to quality control through a further listening of the recording and the correction of any errors. Finally, the interviews were translated into English for publication.

In order to provide concrete findings, data sources and analyst triangulations were carried out (17): the information gathered was verified and compared at different points by means of the field notes collected throughout the training programme. Additionally, analysis was undertaken independently by three researchers, allowing for the identification and management of discrepancies within the codification.

### Ethical considerations

Ethical clearance for the study protocol was approved by the Ethical Committee of the Health Institute Carlos III (Spain). Informed written consent to use and publish their discourses was obtained from all participants in the study. Personal data were managed according to Spanish legislation (18).

## Results

### Knowledge of the disease

With regard to transmission, participants with a higher level of education were particularly concerned with the vector-borne transmission and its prevalence in rural life. They made specific reference to the dangers of houses made of adobe (sun-dried bricks), beds with straw mattresses, and proximity to animals. Participants were familiar with *T*. *infestans* (commonly known as *vinchuca* in Bolivia), and its link to the transmission of the disease. Nevertheless, little was known specifically about how the parasites entered the blood stream. Even if doubts remained surrounding the possibility of vector-borne transmission in Spain, participants felt that it was unlikely to happen due to colder weather, which prevents the existence of the *vinchuca*. They knew that the disease was not transmitted person-to-person. They were also aware of the risk of vector-borne transmission and transmission via blood transfusions; however, they hardly mentioned the possibility of transmission due to ingestion.

While I was sleeping, it would bite me and I'd grab it and pull its head off and throw it on the floor … as my house was made of adobe, it was a mud-roofed house and, because of this, there were quite a few vinchucas. (Woman, age 45)

Thanks God it is not contagious. (Woman, age 44)

However, participants with a lower level of education were unaware of the risk of vector-borne transmission when they lived in Bolivia and, at the time of the interview, they believed incorrectly that the disease could be transmitted through interpersonal contact.

Ever since I was little I've known about the vinchuca, it would bite me … because when we are little we don't know whether it causes the disease or not … even now, my mother doesn't know if it causes the disease. (Woman, age 45)

I wonder if I can pass it on to them or something [talking about her children]. I mean, I am always thinking about it and that is why I have my own glass and I want to be the only one who drinks from it …. (Woman, age 33)

With regards to the symptoms, the disease was viewed as something that *affected a lot of people but only showed up in a few and that too after many years*. When the disease presented itself, it caused death due to heart failure or alterations in the intestine.

It is a disease that, sometimes, in most people, does not show up, as people say … And then, in one, in one or two people, it can show up and they can actually die of … of a heart attack, I've been told. (Woman, age 31)

With regards to the diagnosis, they were aware that detection of the disease was made via a blood test. However, others believed, incorrectly, that it could be detected through urine or faeces. Not all of them knew that a specific test for CD was necessary in order to detect infection; they thought that it could be detected through any blood test.

A long time ago, I thought that a simple blood test would be able to detect it […] that is why I tell people that they need to get tested […], I mean, for Chagas. (Woman, age 34)

Regarding treatment, there were doubts about its effectiveness, duration, side effects, and cost. In general, they knew it did not cure the disease but that it could slow it down and that it was more effective in children and also in adults with a less advanced form of the disease. Trust in the treatment was variable and the motivation for following it was based primarily on blind trust. Even when they knew that the treatment was not 100% effective, the majority of them were accepting of it and were convinced that it would work for them (Table 3).

Basically I understood that the drugs that I am going to take are meant to do kind of keep this … parasite in a balloon. It stays there, in the balloon, kind of separated. So it will affect me as little as possible. (Woman, age 34)

**Table 3:** Considerations of participants regarding the effectiveness of treatment

There is a cure	**E1, E8, E9, E10, E11, E13:** Significant trust in the treatment.
Treatment does not cure; it slows it down or has other benefits	**E2:** (After receiving information) It reduces vertical transmission, it slows down the disease. Is there a cure?
	**E4:** If the disease is not advanced, treatment isolates it, like putting it in a balloon, so it cannot affect me.
	**E5, E7:** (After receiving information) It slows it down so that it is not transmitted from generation to generation.
	**E6:** It can be remedial, in some people it can slow down the disease.
	**E12, E14:** A lot of uncertainty about whether the treatment can cure the disease.
There is no cure	**E2, E3:** (Once diagnosed) There is no cure, it is not worth getting tested.

There is a lack of criticism by some participants regarding the limited visibility of CD. Despite the fact that the treatment is very old and that there have been no new drugs created to treat it, they declared that those currently available must be good enough.

The only thing is that the treatment has been around a long time […] since 1969 and then they came up with this new one … and after this one, no … they haven't brought out any new treatment […]. It must be good! (Woman, age 44)

### Concerns and consequences of the disease

#### Awareness of CD

**Fig. 1:**
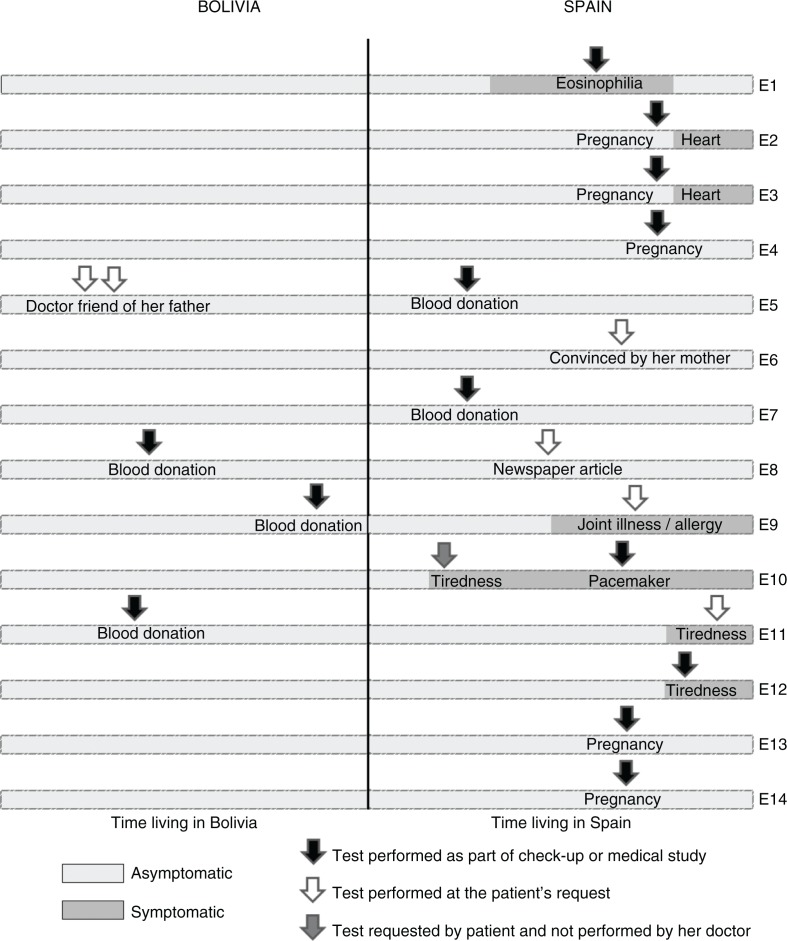
Moment of testing and reason as to why participants underwent Chagas testing.

Most of the participants were diagnosed during a routine medical check-up, pregnancy check-up, or blood donation, and had no perception of the risk at the point of the diagnosis. It is surprising that in some cases the individual had tested positive for Chagas in Bolivia but had not paid much attention to the diagnosis at the time. In other cases, they were the ones who took the initiative of asking for the test (Fig. 1). In these cases, the most important factor in becoming aware of the risk of the disease was to have witnessed the death or illness of a close relative. The decision to get tested was also determined by whether they were aware that treatment was available that could slow down the progression of the disease. Moreover, a woman explained that an article in a Latin American newspaper talking about CD had changed her perception of the risks.

It was about 19 years ago … my oldest son had an accident and needed a blood transfusion from me. It was at that time that I realised I had Chagas […] and the truth is that back in Bolivia, Chagas was seen as nothing major. […] No, there wasn't that much alarm at that time […] That was it! I never got to go to … the centre to do … for them to confirm […] I forgot about it later on! […] When I arrived in Spain […], I happened to glance at a Latin American newspaper and, there you go … and then I felt a bit scared … they called it the ‘silent disease’… a lot of people die in Bolivia because of this disease, which is not considered very important … but they encourage people to go […] to get tested. And I didn't hesitate! I didn't hesitate … I went to do the test and I tested positive. (Woman, age 50)

#### The experience of living with CD and its consequences

When the women were informed about the Chagas diagnosis, different types of feelings arose, ranging from concern to indifference.

It didn't affect me … No, because I didn't know what disease it was. I believed it was something that didn't … something that had a cure … I didn't really know what … what it really was. (Woman, age 31)

The only thing that she told me was, ‘Ah but … back there everyone has Chagas and it's OK.’ (Woman, age 34)

Amongst other women with symptoms or with more information, there was concern about reduced life expectancy and heart problems. Concerns of the participants were mostly surrounding possibly having passed the disease on to their children and the worry of not being able to look after them in the future.

When they told me I had Chagas, I thought that by the time I'm my mother's age … I am also going to be like this, I won't be able to look after my children. […] That's something that scares me. (Triangular Group 1)

The experience of the treatment was negative or positive depending on whether or not there were side effects. In some cases, these side effects were very important.

What's more, you feel powerless because […] they prescribed me the treatment and […] I couldn't even manage to do half of it. Then, to know that … my body has refused the treatment, it also bothers me, right? It bothers me because I've taken so much medicine my whole life […] I am not allergic to any medicine. That's weird, right? (Triangular Group 1)

The motivation to begin treatment was linked to their children, the level of concern they had for the disease, and to the level of trust in the treatment. Additionally, the treatment was perceived as the only option that would improve their chances of living long enough to see their dreams and ambitions come true.

Your only alternatives are to take the treatment or not to take anything. If you don't take anything, it is worse because you are not taking any action. If you are going to take the treatment, you have a chance, and even more being here (Spain), where you are monitored, where you can … you have the support of the doctors. (Triangular Group 2)

However, they also expressed reasons not to seek treatment. These were linked to uncertainty surrounding its effectiveness, side effects, supply shortages, not having anyone to leave their children with, and not having enough time to attend check-ups.

There weren't going to allow me to go to. I don't know how many doctors … And that is the reason why I didn't … I left it. (Woman, age 31)

Another consequence of the disease was the fear of being shunned by Spaniards and by those Latin Americans who were unaware of CD and its mode of transmission. This explains why all the women who were interviewed revealed that they were hiding the disease, especially in their work environment. Nevertheless, they were less concerned about other people from Bolivia finding out, because they felt that Bolivians did know about the disease.

Among our people, I mean, those who really are ‘us’ – that is, the Bolivians – they know the disease, so, there is no problem if you want to tell them … they accept you as you are. We know, more or less … I mean, at least we have an idea of what the disease is, right? We know it isn't contagious or anything. Eh … Ah … I mean, contagious … by talking to people or by touching someone, it isn't contagious but, when talking to other kinds of people who don't have the same nationality as you, then … at least that is what I have felt, you know? (Triangular Group 1)

They were also afraid of being rejected by people from Bolivia due to the connection between CD and poverty.

Every time I have spoken about Chagas, people tell me … Actually, I think they have this idea, like … [clicking sound] … ‘that [happens] to you [Bolivians], because you are so poor.’ (Woman, age 28)

The link between poverty and the disease was evident in the discussions, particularly in the way the women attempted to distance themselves from any connection with a poor or rural background. They would try to look for another explanation for their own infection such as having contracted the disease through their mothers during pregnancy.

The old one [house], yes, that was made of adobe […]. We are from the city, always from the city centre […] And I wonder how I could've been bitten by one of those. I was living in the city, I mean, I have always been from the city. And I don't have any relatives in the countryside, right? Because my grandmother was from the city, from the city centre […]. Since I found out that my mother has it [Chagas], I think it was her who gave it to me during pregnancy. (Woman, age 47)

Participants described instances of being the object of discrimination following the disclosure of their CD. For example, a participant revealed her diagnosis to her house mates and, within a couple of days, they left the house without giving any explanation. Another woman recounted that colleagues at her place of work had separated the kitchen utensils she had been using from their own.

Because the mistake I made was to tell people at work last year … and, my colleagues, they were so ignorant that they even put the glasses aside. (Woman, age 44)

### The social representation of the disease

Participants acknowledged that they came from a region where the *vinchuca* lives and where CD is endemic. This was part of their everyday environment and they had a perception of CD as something common, something that a lot of people had but rarely suffered from. They believed that only some people experience symptoms in later life.

Her grandmother is around sixty or seventy years old and she is very well; and she has that disease. (Woman, age 31)

However, there was a contrasting discourse focused on a fear of Chagas, which was largely the result of having been close to a person with the disease, or to someone who had died suddenly due to cardiac problems.

Everybody has that disease and nothing happens … And I say, OK but my mother has it and she is having a bad time. (Woman, age 34)

While there was a sense of indifference towards the disease prior to the diagnosis, there was a sudden association with death upon confirmation of the infection. This was evident in the experiences of the participants as well as those of their families.

I feel very bad. No, actually I was fine when this disease wasn't here, I was fine, now it is here and I feel bad. I feel bad. **What makes you feel bad?** Because maybe I am going to die [she cries] […] I don't know what I am going to do, sometimes I think that I am going to die at any moment. (Woman, age 45)

Women tend to protect themselves, thinking it is better not to know of the diagnosis as if they felt that being aware of the disease would make it *grow*.

You feel healthy and then someone tells you that you have something. Everything is psychological too so, by saying it, you get ill straight away. It is better not to know and not to worry […]. If you think about it, you draw it towards you. And that is why people say it is better not to know. (Woman, age 42)

Interestingly, participants considered that people from Bolivia were irresponsible and careless with regards to their own health. Nevertheless, they pointed out that if the healthcare system in Bolivia were free and of good quality, they would look for healthcare assistance.

If the treatment were free, or even if you had to pay a little, but not much, people would do it […] [people] would care [about their own health] but only if it does not cost a lot of money. (Woman, age 42)

## Discussion

### Knowledge

The interviewees are highly familiar with vector-born transmission as it is endemic to the regions they are from (19, 20). However, as has already been reported in other research studies conducted in endemic and non-endemic areas, they have a poorer understanding of the symptoms, the diagnosis, the transmission routes and the treatment (12, 21). These gaps in their knowledge are not only related to their level of education but may also be explained by the difficulties that arise surrounding doctor–patient communication or the lack of awareness of the disease among healthcare workers (22). As the women who participated in this study had repeatedly visited healthcare services in Spain to treat their CD, their lack of knowledge could be related to the lack of information provided by these professionals. Therefore, it is very important that people working in healthcare services develop their awareness of the disease and its social consequences. Furthermore, they should possess the necessary tools to be able to give effective information to their patients.

Significantly, we found that there is confusion surrounding treatment. This seems understandable given the high level of uncertainty that currently exists regarding its effectiveness. Therefore, public health campaigns need to communicate clearly the positive outcomes in the prevention and control of congenital CD (23). Given that the biggest concern for our participants was their children, these messages could have a very positive effect on the wider population infected by *T. cruzi*.

It is interesting to highlight the absence of criticism surrounding the limited visibility of CD and of the drugs that are currently available. The participants never mentioned the inefficiencies of the government in managing the disease or the pharmaceutical industry's general lack of interest in researching for new drugs (24). These factors are common in neglected diseases whereby the affected population is characterised by an inability to react (24, 25).

### Concerns and consequences of the disease

One of the most important findings from this study is the women's limited understanding of the risks associated with CD before being diagnosed. The fact that people remain asymptomatic for a long period of time could explain this, as well as the lack of awareness of the disease (11, 26).

Our data provide evidence of the close relationship between lack of awareness of the disease and the social representation of CD in Bolivia, where it is perceived as a common and even relatively harmless condition. Consequently, we would expect that people will act in accordance to this social representation (27) and that it could be a determining factor in Bolivian migrants being less likely to access healthcare facilities and to ask for a screening test.

Other explanations behind the lack of awareness of the disease amongst women from urban areas of Bolivia could lie in the fact that they relate Chagas to their conception of rural life. This idea could mistakenly lead people to associate the disease with rural areas. This could in turn make people from urban areas feel an illusionary invulnerability to the disease and consequently refrain from taking preventive measures (21). Currently, a high percentage of Chagas cases are diagnosed in Bolivian cities (28, 29).

The low level of risk awareness that is reported in this study suggests the need to implement health education campaigns about the risk factors for acquiring the disease and the importance of early diagnosis. At present, there are few health education initiatives in Spain and those that do exist are only at a local level (30).

Our research provides evidence that the biggest concerns felt by the women – both at the time of the diagnosis and afterwards – surround their children. As other studies have already demonstrated (10, 31), a sense of responsibility and guilt appears to be caused by the fear of having passed the disease on to their children and of not being able to look after them if they become ill.

Concern and uncertainty surrounding treatment was also reported. The interviewed women gave reasons for not adhering to treatment which have also been well documented in the wider research. These included the poor tolerance of drugs in adults (32, 33) or the time that follow-up appointments take, resulting in the need to be absent from work or not having anyone to look after their children (31).

Our study shows that there are cases of discrimination. It was observed that the affected individuals tended to hide the disease, fearing rejection and its consequences, such as social isolation and the loss of employment (24, 25, 34–37). The lack of awareness of the disease, fear of being infected, and the prevalent association with dirt, misery and rural areas were the most relevant reasons for discrimination and rejection (25, 37, 38). Moreover, the high prevalence of CD in Bolivia may lead to stereotypes in certain healthcare settings, like the ‘Chagasic Bolivian’ (11). All these elements could be undermining the development of a positive perception of what it is like to live with Chagas.

### Representation of the disease

This study has employed a transnational perspective (39) which has been a key factor in identifying, understanding, and explaining the complexity of the multiple interrelations that exist surrounding the social representation of CD (40). Evidence shows that immigrants build their experiences and perceptions through their own past and the multiple relationships they previously had in their country of origin (familial, social, economic, and religious). These perceptions cross geographical, cultural, and political borders between both countries (Bolivia–Spain).

Our data show how the social representation of the disease ranges in a *continuum* from a common disease to a deadly disease. Absence of symptoms together with the lack of impact on day-to-day activities has contributed to the normalisation of the illness (37, 40, 41) to the point where Chagas is not considered a disease (25).

The standard of living in Bolivia over the past 50 years may have influenced the perception of the disease. Until a decade ago, it was endemic but silent. The country had an extremely high rate of households infested with *T*. *infestans* and a high percentage of the population was undiagnosed yet infected and asymptomatic. Moreover, the access to testing or specific treatment was very limited. Likewise, healthcare policies and the media have been busy dealing with more urgent and visible problems (natural disasters and epidemics), the result of which is that the disease has been largely forgotten about (24, 42). Due to the circumstances highlighted above and given the country's low life expectancy, the disease has not reached social relevance because individuals with the Chagas infection are likely to have died from natural causes at the age when the disease would most likely have presented itself (25, 37). Nonetheless, during the past few years, the standard of living has generally improved and life expectancy has risen considerably (43). Therefore, in this new scenario, people infected with Chagas are middle-aged adults who are productive members of society. As such, it is feared that the disease may result in huge economic losses and the undermining of personal ambitions. This is the reason why both governments and society as a whole are starting to attribute importance to the disease and to tackle the situation by carrying out the fumigation of houses and providing better access to treatment (43).

The normalisation and lack of visibility of the disease is in juxtaposition with its association with death (10, 11, 31). In countries where the healthcare system is largely private, people generally tend to resort to home remedies and traditional medicine until they are very unwell and nothing can be done for them, thus creating an association with death in the collective memory (31, 44, 45). This would explain the gap between the collective construction of the natural history of the disease and its reality. Therefore, the increase in information surrounding CD and the availability of an early diagnosis would most likely help to alter the perception of the disease (10, 31).

Finally, thought patterns introducing the influence of the *psychological* on the *biological* that maintain ‘the less you know, the less you suffer’ are common amongst Bolivian migrants (10). This can create a sizeable obstacle in reaching this population through public health programmes.

### Strengths and limitations

The exclusion of men in the study is a limitation as it leads to a partial understanding of what living with CD means for the Bolivian population residing in Madrid. However, the inclusion of only women is also a strength given that the control of vertical transmission during pregnancy is one of the main priorities in non-endemic countries.

## Conclusions

Migratory flows create new public health needs and challenges, as is the case with CD in Spain. Programmes and healthcare providers need to take into account how the affected persons think, how they act, and what their life experiences are. This could improve both the control of the disease and the quality of life of those affected (40).
